# Status of infection prevention and control programs in 25 facilities of Rwanda: Results from the WHO infection prevention and control assessment framework

**DOI:** 10.1002/puh2.183

**Published:** 2024-05-19

**Authors:** Jean Jacques Irakiza, Christian Mazimpaka, Dieudonne Ndatimana, John Baptist Kalach, Vincent Hatangimbabazi, Edouard Kamuhangire, Alphonsine Mukamunana, Olive Ntakirutimana, Joseline Tengera, Olivier Ruhumuriza, Onesime Manishimwe, Assumpta Kayinamura Mwali, Erigene Rutayisire

**Affiliations:** ^1^ IntraHealth International Kigali Rwanda; ^2^ Ministry of Health Kigali Rwanda; ^3^ College of Medicine and Health Sciences School of Public Health University of Rwanda Kigali Rwanda

**Keywords:** Africa, healthcare‐associated infections, infection prevention and control, Infection Prevention and Control Assessment Framework, Rwanda, Sub‐Saharan Africa, World Health Organization

## Abstract

**Background:**

Infection prevention and control (IPC) is important in ensuring patient safety, protecting healthcare workers, and reducing healthcare‐associated costs. The World Health Organization (WHO)‐validated Infection Prevention and Control Assessment Framework (IPCAF) was used to evaluate IPC practices in Rwandan healthcare facilities.

**Methodology:**

In this cross‐sectional study, we assessed 25 health facilities across Rwanda, including district and referral hospitals. Using the IPCAF tool, we assessed eight core components (CCs) of IPC programs. We calculated median scores and interquartile ranges to determine the levels of implementation of IPC practices.

**Findings:**

Among the 25 facilities, all showed some degree of IPCAF implementation, with an overall median IPCAF score of 545.0, reflecting an intermediate level. Three facilities (12%) were at a basic level, 16 (64%) at an intermediate level, and 6 (24%) at an advanced level of IPC practices. The presence of IPC guidelines scored the highest among CCs (median: 87.5). About 96% of facilities did not have a dedicated full‐time IPC staff, 64% of facilities did not offer IPC training to new staff, and 84% did not have protocols for multidrug‐resistant pathogens.

**Conclusions:**

This initial IPCAF assessment in Rwanda reveals critical IPC strengths and gaps. These findings highlight the necessity for targeted interventions, such as appointing dedicated IPC staff, strengthening IPC committees, and enhancing IPC training and resources.

## Graphical abstract

The assessment of the eight IPC core components revealed varying levels of implementation across the facilities. The most performing core component was IPC guidelines (CC2) with a median score of 87.5. The least performing core component was workload, staffing, and bed occupancy (CC7) with a median score of 50.0

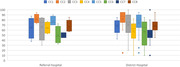

## INTRODUCTION

Hospital‐acquired infections (HAIs) are a significant global health issue, contributing to patient morbidity and mortality. They affect an estimated 1.4 million individuals globally and are associated with approximately 100,000 fatalities each year [1, 2]. In low‐ and middle‐income countries (LMICs), the prevalence of HAIs is reportedly 25%, a contrast to the 7% prevalence noted in high‐income nations [2, 3, 4]. The high prevalence of HAIs in LMICs heightens the burden on health systems, leads to extended hospital stays, increases workloads for healthcare providers, escalates antimicrobial resistance (AMR), and increases costs on the health system [5, 6, 7].

In Rwanda, the rate of HAIs is estimated at 32%. There is a significant prevalence of AMR among the main causative agents of infections. Notably, 42% of *Escherichia coli*, 18% of *Staphylococcus aureus*, and 15% of *Klebsiella* species display resistance. Furthermore, a 74% resistance rate to third‐generation cephalosporins has been documented [8]. This increase in AMR poses a significant challenge to the country's healthcare system, affecting infectious disease management and control. Therefore, the implementation of robust infection prevention and control (IPC) measures is critical in fostering patient safety and attenuating the morbidity and mortality rates associated with HAIs [9].

The IPC framework within Rwandan healthcare systems is characterized by the integration of various policies and strategic approaches, including the national hospital accreditation standards [10]. These standards serve as a benchmark for excellence and safety in healthcare delivery across the health sector. This commitment to high standards of IPC is mirrored across individual health facilities, where efforts are made to develop and implement policies and procedures that not only comply with national accreditation criteria but also are tailored to the unique operational contexts of each facility.

However, a comprehensive assessment of the implementation and effectiveness of these programs using the Infection Prevention and Control Assessment Framework (IPCAF) tool has not yet been conducted. This study aimed to evaluate the status of IPC program implementation across a variety of Rwandan health facilities using the World Health Organization (WHO)‐validated IPCAF tool [11]. This tool is designed to improve the effectiveness of IPC programs through baseline and continuous assessments. It assesses the current IPC landscape at the targeted facility, explores existing IPC activities and resources, and pinpoints areas of strength as well as gaps necessitating improvement, thereby informing the development of action plans for enhanced IPC implementation.

## METHODS

### Study design and population

This study employed a cross‐sectional design to assess 25 health facilities across Rwanda from June to September 2022. The facilities were selected using a stratified random sampling technique, which assured representation from all 5 provinces of the country and included 4 referral hospitals and 21 district hospitals. The stratification of facilities was conducted based on their respective levels, with a predetermined number being randomly selected from each stratum to undergo the IPCAF assessment.

### Study setting and intervention

This assessment was conducted across 25 health facilities across the country, including 21 district hospitals and 4 tertiary healthcare facilities or referral hospitals. There are in total 40 district hospitals across the country that primarily serve primary care patients referred from health centers and do not offer microbiology laboratory services, as well as 12 referral hospitals across the country that offer specialized services for patients referred from district hospitals and offer microbiology laboratory services [12].

### Data collection and analysis

The data collection was conducted by the IntraHealth International IPC technical team, which underwent training on the utilization of the IPCAF tool. The team executed the assessment by conducting interviews with the IPC focal person allocated at each facility. The evaluation of IPC practices was done through observation, interviews with key personnel, and a review of documents during the site visits. All data collection was done using paper‐based methods. Data were captured and analyzed using Stata version 17. The analysis described the demographic capacity of assessed hospitals, the distribution of assessed facilities, median IPCAF scores, and compliance levels by facility type (*N* = 25). IPC compliance levels were categorized based on the median IPCAF scores.

### Infection Prevention and Control Assessment Framework (IPCAF)

The IPCAF is partitioned into eight sections. Each section corresponds to one of the WHO's core components (CCs) of IPC programs. These CCs present a comprehensive framework for evaluating the IPC practices within each health facility. The IPCAF tool enables healthcare facilities of varying settings to conduct IPC self‐assessments on the implementation of IPC CCs to identify gaps and areas for improvement [11].

Each of the eight CCs has a maximum score of 100 points, with the highest possible overall IPCAF score being 800 points. IPCAF total scores are classified into four levels—Inadequate (0–200) indicating significant IPC implementation gaps, Basic (201–400) indicating some IPC functions but lacking proper implementation, Intermediate (401–600) indicating that most IPC aspects are properly implemented, and Advanced (601–800) corresponding to full IPC implementation according to WHO standards (Table 1).

**TABLE 1:** Infection Prevention and Control Assessment Framework (IPCAF) tool scores and description.

IPCAF scores	Level	Description
0–200	Inadequate	Insufficient IPC implementation and require significant improvements in almost all components
201–400	Basic	These facilities have some functional IPC aspects without proper implementation, and additional improvement is needed
401–600	Intermediate	At this level, most IPC aspects are properly implemented
601–800	Advanced	At this level, the IPCs are fully implemented according to WHO recommendations

Abbreviation: IPC, Infection prevention and control.

#### CC1: IPC program

This part assessed the availability of an operational IPC program. It assessed whether a facility has clearly defined objectives targeting the prevention of HAIs and combating AMR, with designated staff and a committee at the facility level responsible for these objectives.

#### CC2: IPC guidelines

This part assessed the availability and adoption of evidence‐based IPC guidelines tailored to the facility's needs. It also assessed whether a facility conducted the training of healthcare workers on the guidelines and monitored the guidelines implementation.

#### CC3: IPC education and training

This part assessed the presence of task‐based IPC education and training strategies. It also assessed whether the strategies involved skilled and trained staff at the health facility level, including simulation and bedside training.

#### CC4: HAI surveillance

This part assessed the health facility's surveillance system for HAIs. It assessed whether the system facilitates early outbreak detection and the provision of IPC priority interventions, as well as feedback to healthcare workers on infection status at the facility level.

#### CC5: Multimodal strategies for implementation of IPC interventions

This part assessed the availability of tools and checklists, developed by multidisciplinary teams at the facility, for preventing and controlling HAI and AMR. It also evaluated whether these tools and checklists incorporated the five components of multimodal strategies, namely, system change, education and training, monitoring and feedback, communication and reminder, safety climate, and culture change.

#### CC6: Monitoring/audit of IPC practices and feedback

This part evaluated the frequency and regularity of IPC practice audits and monitoring, including the competency of the staff and the tools employed in these processes.

#### CC7: Workload, staffing, and bed occupancy

This part focused on staffing and bed occupancy, comparing the facility's conditions against WHO standards.

#### CC8: Environments, materials, and equipment for IPC

This part assessed the environmental safety factors for preventing and controlling HAI and AMR during patient care. These factors included water supply, hand hygiene and sanitation infrastructure, power supply, ventilation and cleaning, availability of personal protective equipment (PPE), patient placement, medical waste management and sewages and decontamination and sterilization protocols.

## RESULTS

The study evaluated 21 out of 40 district hospitals and 4 out of 12 referral hospitals in Rwanda. The four referral hospitals had a median IPCAF score of 532.5, corresponding to an intermediate IPC level. The 21 district hospitals had a median IPCAF score of 545, also corresponding to an intermediate IPC level (Table 2).

**TABLE 2:** Distribution of facility assessed, median Infection Prevention and Control Assessment Framework (IPCAF) scores, and levels by facility type (*N* = 30).

Facility type	*N*	%	Median	IQR	Level
Tertiary health care	4	16.0	532.5	(456.9, 585.6)	Intermediate
Secondary health care	21	84.0	545	(481.8, 612.3)	Intermediate
**Total**	**25**	**100.0**	**545**	**(481.8, 598.5)**	**Intermediate**

Abbreviation: IQR, interquartile range.

Out of 25 facilities, none were inadequate in IPC CCs. Three facilities (12%) showed basic implementation, needing further improvements. Sixteen facilities (64%) reached an intermediate level, and six facilities (24%) achieved advanced implementation (Table 3).

**TABLE 3:** Distribution of assessed facilities by Infection Prevention and Control Assessment Framework (IPCAF) levels (*N* = 25).

IPCAF score	Category	*N*	%
0–200	Inadequate	0	0.0
201–400	Basic	3	12.0
401–600	Intermediate	16	64.0
601–800	Advanced	6	24.0

Abbreviations: IPC, infection prevention and control; WHO, World Health Organization.

**FIGURE 1:**
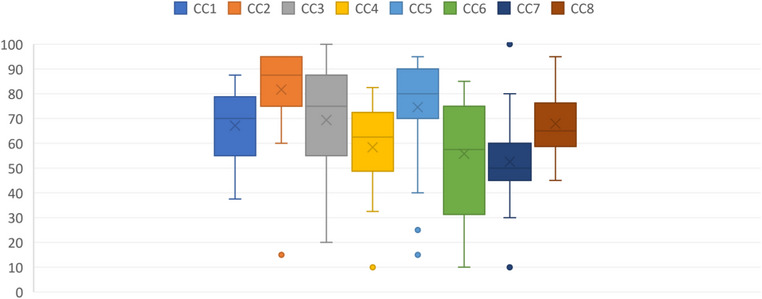
(A) The distribution of Infection Prevention and Control Assessment Framework (IPCAF) median scores of the eight core components (*N*: 25). The boxes represent the interquartile range (IQR), including the median, which is represented by the middle horizontal line. The whiskers represent the full range, and the dots represent the outliers. (B) The distribution of IPCAF median scores of the eight core components, segregated by type of facility type (*N*: 25). The boxes represent the IQR, including the median, which is represented by the middle horizontal line. The whiskers represent the full range, and the dots represent the outliers.

The assessment of the eight IPC CCs revealed varying levels of implementation across the facilities. The most positively performing CCs were IPC guidelines (CC2) with a median score of 87.5. The CCs with the lowest median scores were workload, staffing, and bed occupancy (CC7) at 50.0 and monitoring/audit of IPC practices and feedback (CC6) at 57.5 (Figure 1A,B).

### Presence of an IPC program staff (CC1)

Out of the 25 assessed health facilities, 18 (72%) had an IPC program with clearly defined objectives and an annual activity plan, with a median score of 70.0 (interquartile range [IQR]: 55.0, 78.8). IPC committees were present in 22 (88%) facilities, with 14 (56%) of them having a member of the hospital senior leadership staff involved. In 14 (56%) of these facilities, the IPC committees had clearly defined objectives, indicators, and future targets. Institutional support for budgeting was available in 20 (80%) facilities and was available for meetings in 19 (76%) facilities (Table 4).

**TABLE 4:** Key findings of Infection Prevention and Control Assessment Framework (IPCAF) assessment in selected health facilities in Rwanda.

Core components	Indicators	Frequency (*N* = 25)	%
CC1: IPC program			
IPC program	IPC program with clearly defined objectives and annual activity plan	18	72
	IPC program without clearly defined objectives and annual activity plan	7	28
	IPC team comprising IPC professionals	19	76
	At least one full‐time IPC professional or equivalent to <250 beds	1	4
	IPC team or focal person has dedicated time for IPC activities	21	84
	IPC team include both doctors and nurses	17	68
IPC committee			
	IPC committee actively supporting the IPC team	22	88
	Senior facility leadership in IPC committee	14	56
	Senior clinical staff	25	100
	Facility management	24	96
	IPC clearly defined objectives, indicators, and set future targets	14	56
Institution support			
	Senior facility leadership shows clear commitment and support; budget	20	80
	Senior facility leadership shows clear commitment and support; meeting rounds	19	76
	Microbiological laboratory support for routine day‐to‐day use	0	0
CC2: IPC guideline			
Available guideline for			
	Standard precautions	19	76
	Hand hygiene	23	92
	Transmission‐based precautions	15	60
	Outbreak management and preparedness	23	92
	Prevention of surgical site infection	21	84
	Prevention of vascular catheter‐associated bloodstream infections	22	88
	Prevention of hospital‐acquired pneumonia (HAP); all types of HAP, including (but not exclusively) ventilator‐associated pneumonia	7	28
	Prevention of catheter‐associated urinary tract infections	21	84
	Prevention of transmission of multidrug‐resistant (MDR) pathogens	4	16
	Disinfection and sterilization	25	100
	Healthcare worker protection and safety	17	68
	Injection safety	20	80
	Waste management	24	96
	Antibiotic stewardship	5	20
Guideline development and monitor			
	Guidelines are consistent with national/international policies	24	96
	Relevant stakeholders involved in the development and adaptation of the IPC guidelines, in addition to IPC personnel	23	92
	Healthcare workers receive specific training related to new or updated IPC guidelines introduced in the facility	22	88
	Healthcare workers regularly monitor the implementation of at least some of the IPC guidelines in your facility	20	80
CC3: IPC education and training			
IPC training	Personnel with the IPC expertise to lead IPC training	21	84
	New employee orientation and regular mandatory IPC training	9	36
	Additional non‐IPC personnel with adequate skills to serve as trainers and mentors	21	84
	New employee orientation and regular mandatory for others	14	56
	Does administrative and managerial staff receive general training regarding IPC in your facility	19	76
	Has specific IPC training for patients or family members to minimize the potential for HAIs	18	72
Evaluation of IPC training			
	Has periodic evaluations of the effectiveness of training programs, regularly	7	28
	Ongoing development/education offered for IPC staff	14	56
CC4: Surveillance			
Organization of surveillance			
	Defined component IPC program surveillance	21	84
	Presence of a personnel responsible for surveillance activities	23	92
	Have the professionals responsible for surveillance activities been trained in basic epidemiology, surveillance, and IPC	20	80
	Presence of informatics/IT support to conduct your surveillance	10	40
Priority of surveillance and conducting area			
	Do you go through a prioritization exercise to determine the HAIs to be targeted for surveillance according to the local context?	21	84
	Surgical site infections	24	96
	Device‐associated infections	7	28
	Clinically defined infections	17	68
	Colonization or infections caused by MDR pathogens, according to your local epidemiological situation	1	4
	Local priority epidemic‐prone infections (e.g., norovirus, influenza, tuberculosis [TB], severe acute respiratory syndrome [SARS], Ebola, Lassa fever)	18	72
	Infections in vulnerable populations (e.g., neonates, intensive care units, immunocompromised and burn patients)	18	72
	Infections that may affect healthcare workers in clinical, laboratory, or other settings	8	32
Methods of surveillance			
	Do you regularly evaluate if your surveillance is in line with the current needs and priorities of your facility?	11	44
	Use of reliable surveillance case definitions	16	64
	Use of standardized data collection methods according to international surveillance protocols or, if adapted, through an evidence‐based adaptation process and expert consultation	12	48
	Have processes in place to regularly review data quality	16	64
	Have adequate microbiology and laboratory capacity to support surveillance and can reliably identify pathogens and antimicrobial drug resistance patterns in a timely manner	1	4
Information analysis, dissemination, and governance			
	Surveillance data are used to make tailored unit/facility‐based plans for the improvement of IPC practices	19	76
	Analyze antimicrobial drug resistance on a regular basis	1	4
	Provide regular feedback on up‐to‐date surveillance information to: frontline healthcare workers	19	76
	Provide regular feedback on up‐to‐date surveillance information to clinical leaders/heads of department	20	80
	Provide regularly feedback on up‐to‐date surveillance information to clinical leaders/heads of department IPC committee	20	80
	Provide regularly feedback on up‐to‐date surveillance information to nonclinical management/administration	17	68
	Feedback on up‐to‐date surveillance information is provided through presentation and interactive problem‐orientated solution findings	17	68
CC5: Multimodal strategies			
Multimodal element inclusions			
	Use multimodal strategies to implement IPC interventions	23	92
	System change: intervention to ensure the necessary infrastructure and continuous availability of supplies are in place	17	68
	Education and training: written information and/or oral instruction and/or eLearning only	5	20
	Monitoring and feedback: monitoring compliance with process or outcome indicators	13	52
	Communication and reminder: reminders, posters, or other advocacy/awareness‐raising tools to promote the intervention	21	84
	Safety climate and culture: managers/leaders show visible support and act as champions and role models, promoting an adaptive approach and strengthening a culture that supports IPC, patient safety, and quality	16	64
Implementation strategy	Strategies include bundles or checklists	18	72
	Do you regularly link to colleagues from quality improvement and patient safety to develop and promote IPC multimodal strategies?	22	88
	Is a multidisciplinary team used to implement IPC multimodal strategies?	22	88
CC6: Monitoring/audit of IPC practices and Feedback			
Monitoring plan			
	Have trained personnel responsible for monitoring/audit of IPC practices and feedback	25	100
	Don't have a well‐defined monitoring plan with clear goals, targets, and activities	14	56
	Hand hygiene compliance	22	88
	Intravascular catheter insertion and/or care	7	28
	Wound dressing change	3	12
	Transmission‐based precautions and isolation to prevent the spread of multidrug‐resistant organisms (MDRO)	6	24
	Cleaning of the ward environment	16	64
	Disinfection and sterilization of medical equipment/instruments	16	64
	Consumption/Usage of alcohol‐based hand‐rub or soap	11	44
	Consumption/Usage of antimicrobial agents	1	4
	Waste management	20	80
Feedback and auditing report			
	IPC team	12	48
	The WHO hand hygiene self‐assessment framework survey is undertaken annually	15	60
	Assess safety cultural factors in your facility	8	32
CC7: Workload, staffing, and bed occupancy			
Staffing			
	Appropriate staffing levels assessed in your facility according to patient workload using national standards or a standard staffing needs assessment tool such as the WHO Workload Indicators of Staffing Need method	18	72
	An agreed ratio of healthcare workers to patients maintained across your facility for all healthcare workers in the facility	5	20
	A system in place in your facility to act on the results of the staffing needs assessments when staffing levels are deemed to be too low	18	72
Bed occupancy			
	The design of wards in your facility in accordance with international standards regarding bed capacity	4	16
	Bed occupancy in your facility kept to one patient per bed	7	28
	No patients in your facility placed in beds standing in the corridor outside of the room	20	80
	Adequate spacing of >1 m among patient beds ensured	6	24
	A system in place in your facility to assess and respond when adequate bed capacity is exceeded; this is the responsibility of the hospital administration/management	15	60
CC8: Built environment, material, and equipment for IPC at the facility level			
Water			
	Availability of water services at all times and of sufficient quantity for all uses	13	52
	Reliable safe drinking water station present and accessible for staff, patients, and families at all times and in all locations/wards	13	52
Hand hygiene and sanitation facilities			
	Functioning hand hygiene stations available at all points of care	9	36
	More than four toilets or improved latrines available for outpatient settings, or ≥1 per 20 users for inpatient settings, sufficient number present and functioning	7	28
Power supply, ventilation, and cleaning			
	Sufficient energy/power supply available at day and night	23	92
	Functioning environmental ventilation available in patient care areas	19	76
	For floors and horizontal work surfaces, is there an accessible record of cleaning, signed by the cleaners each day? Record exists but is not completed and signed daily	4	16
	Appropriate and well‐maintained materials for cleaning are available	15	60
	Single‐patient rooms or rooms for cohering patients with similar pathogens if the number of isolation rooms is insufficient	16	64
Cohering and PPE use	PPE available at all times and in sufficient quantity for all uses for all healthcare workers	15	60
Medical waste and sewage management	Functional waste collection containers for non‐infectious (general) waste, infectious waste, and sharps waste in close proximity to all waste generation points	19	76
	Functional burial pit/fenced waste dump or municipal pick‐up available for disposal of non‐infectious (nonhazardous/general) waste	14	56
	Presence of incinerator or alternative treatment technology for the treatment of infectious and sharp waste	19	76
	Functional wastewater treatment system available	13	52
Decontamination and sterilization			
	Functional reliably dedicated decontamination area and/or sterile department	16	64
	Have reliable, sterile, and disinfected equipment ready for use	23	92
	Disposable items are continuously available when necessary	22	88

Abbreviation: IPC, infection prevention and control.

### IPC guidelines available (CC2)

The IPC guidelines (CC2) had a high score of 87.5 (IQR: 75.0, 95.0), making it the highest scoring CCs. Disinfection and sterilization guideline was the most available in all assessed facilities at 100%, whereas the guideline for prevention of transmission of multidrug‐resistant (MDR) pathogens was the least available, with only 4 (16%) of assessed facilities having it. More than half of the assessed facilities had guidelines for standard precautions (76%), hand hygiene (92%), transmission‐based precautions (60%), outbreak management and preparedness (92%), and prevention of surgical site infections (84%). Guidelines on antibiotic stewardship and prevention of hospital‐acquired pneumonia were available in 20% and 28% of facilities, respectively. Overall, 96% of hospitals had IPC guidelines consistent with national and international policies, with 92% of facilities their relevant stakeholders involved in guideline development. Healthcare workers from 88% of hospitals had been trained on new and adopted IPC guidelines, whereas 80% of these facilities monitored the implementation of the guidelines by their healthcare workers (Table 4).

### IPC education and training (CC3)

The median score for this CC was 75.0 (IQR: 55.0, 87.5). In 84% of the facilities, IPC training was conducted by trained staff. However, 64% of new employees in the assessed facilities did not receive IPC training or orientation. Administrative and managerial staff from 76% of the facilities underwent IPC training, whereas 72% of facilities provided orientation to patients and their family members. Only 28% of the facilities regularly evaluated their IPC training programs (Table 4).

### HAI surveillance activities (CC4)

The median score for HAI surveillance was 62.5 (IQR: 48.8, 72.5). Surveillance programs were implemented in 84% of the facilities, with 40% utilizing informatics and technology. In 80% of facilities, professional staff with basic epidemiology training were responsible for surveillance activities. Infections caused by MDR pathogens were less surveilled at 4%, whereas surgical site infections were the most surveilled at 96%. Regular evaluations of surveillance occurred in 44% of facilities, with 64% using international or adopted protocols. Only one facility (4%) conducted surveillance based on microbiology laboratory results (Table 4).

### Multimodal strategies (CC5)

In 92% of facilities, a multimodal strategy for implementing IPC was used, with a median score of 80.0 (IQR: 70.0, 90.0). Outcome indicators were monitored in 52% of facilities. Additionally, managers and leaders in 64% of the facilities actively supported and served as role models for safety and climate change initiatives (Table 4).

### Monitoring and audit of IPC practices and feedback (CC6)

CC6 had the widest range, with a median score of 57.5 (IQR: 31.0, 75.0). Although all facilities (100%) had trained staff to monitor and audit IPC activities and provide feedback to all staff, only 44% had a well‐defined monitoring plan with clear objectives and targets. Certain indicators were monitored more frequently than others: hand hygiene (88%), waste management (80%), and disinfection and sterilization along with cleaning (both at 64%). Other indicators were monitored in less than a third of the assessed health facilities. The WHO hand hygiene self‐assessment was conducted regularly in 60% of the facilities (Table 4).

### Workload, staffing, and bed occupancy (CC7)

The lowest median score was found in workload, staffing, and bed occupancy at 50.0 (IQR: 45.0, 60.0). Staffing levels according to patient workload were assessed in 72% of facilities. The patient‐to‐healthcare worker ratio was maintained in only 20% of facilities. Bed arrangements based on international standards were present in 16% of facilities. Bed spacing of less than 1 m was observed in 24% of facilities (Table 4).

### Environment, materials, and equipment for IPC (CC8)

The median score for the environment, materials, and equipment was 65.0 (IQR: 58.8, 76.3). Sufficient water for safe drinking and accessibility was available in 52% of facilities for general use. Functional handwashing stations and improved latrines were present in 36% of facilities. A sufficient power supply was available in 92% of facilities, whereas proper ventilation was found in 76%. Cleaning records were present but unsigned and outdated in 16% of facilities. PPE for healthcare providers and cleaners was available in 60% of facilities, and waste containers for different waste types were present in more than half of the health facilities (Table 4).

## DISCUSSION

This study presents a comprehensive assessment of IPC practices across Rwanda's public healthcare facilities, including both district and referral hospitals. The findings offer a nuanced view of the IPC landscape, revealing an overall intermediate level of IPC implementation across the evaluated facilities. This intermediate level of implementation is promising, as none of the facilities were rated inadequate in core IPC components. However, the variation in implementation levels across different CCs underscores the complexity of achieving high IPC standards and highlights specific areas for improvement.

The relatively high scores in IPC guidelines (CC2) and the presence of IPC program staff (CC1) in a majority of the facilities reflect a strong foundational understanding and commitment to IPC standards. These components are crucial for establishing a culture of IPC within healthcare settings. The widespread availability of disinfection and sterilization guidelines and the institutional support for IPC activities indicate a prioritization of these areas. Nevertheless, the limited availability of guidelines for the prevention of transmission of MDR pathogens in only 16% of assessed facilities points to a significant gap that needs addressing, especially in the context of rising global AMR [7, 8, 13]. The study also identifies critical areas requiring attention and improvement, notably in workload, staffing, and bed occupancy (CC7) and in the monitoring/audit of IPC practices and feedback (CC6). These components scored the lowest among the eight CCs, highlighting challenges in maintaining adequate staffing levels and in effectively monitoring IPC practices. The suboptimal scores in these areas suggest a need for increased staffing and better designed monitoring systems that could enhance IPC implementation levels [14].

Education and training (CC3) were another area with room for improvement. Although a majority of facilities conduct IPC training, the lack of orientation for new employees and regular evaluation of IPC training programs highlights a missed opportunity for reinforcing IPC practices among all healthcare workers. This finding is consistent with other studies that emphasize the importance of comprehensive training programs in improving IPC practices and outcomes [15, 16]. Additionally, the variability in HAI surveillance activities (CC4) and the less frequent surveillance of infections caused by MDR pathogens represent a critical area for strengthening efforts to identify and manage HAIs more effectively. Enhancing surveillance activities could lead to better outbreak management and a reduction in HAIs across healthcare facilities [17].

The Rwanda Health Sector annual performance report for 2021 showed that the country has 55 public hospitals, including 8 national referral hospitals, 8 provincial hospitals, and 39 district hospitals [18]. Assessing IPC practices in 25 public hospitals (45.4% of all public hospitals in Rwanda), including referral and district hospitals, using WHO standard IPCAF tools is the main strength of this study. Furthermore, using well‐trained IPC personnel during the assessment improves the reliability of our results. To our knowledge, this is the first national assessment of IPC practices in public hospitals in Rwanda. We also acknowledge some limitations such as using the same IPCAF tool to assess IPC practices in district and referral hospitals. However, this may not potentially affect the results, as only four referral hospitals were included in the study, and a subanalysis was performed to understand the IPC practices in both district and referral hospitals.

In conclusion, this inaugural IPCAF assessment of IPC status at the facility level in Rwanda has shed light on the strengths, challenges, and opportunities in enhancing and institutionalizing IPC measures. The findings reveal an intermediate level of IPC implementation across Rwandan public healthcare facilities, with areas of both strength and opportunities for improvement. Strengthening the weakest links, particularly in workload management, staff training, and the monitoring of IPC practices, as well as enhancing guidelines for emerging threats like MDR pathogens, could propel these facilities toward advanced IPC levels. As IPC remains a critical component of healthcare quality and patient safety, targeted interventions based on these findings could significantly contribute toward IPCAF at an advanced level for all facilities and eventually lead to healthcare outcomes improvement in Rwanda.

## AUTHOR CONTRIBUTIONS

Irakiza Jean Jacques, Christian Mazimpaka, Assumpta Kayinamura Mwali, and Erigene Rutayisire led the conception, design, and implementation of the study, as well as the literature review, data analysis, and manuscript writing. Dieudonne Ndatimana, John Baptist Kalach, Vincent Hatangimbabazi, Edouard Kamuhangire, Alphonsine Mukamunana, Olive Ntakirutimana, Joseline Tengera, Olivier Ruhumuriza, and Onesime Manishimwe supported data interpretation and manuscript writing. All authors reviewed and approved the final manuscript.

## CONFLICT OF INTEREST STATEMENT

The authors declare that they have no conflicts of interest.

## FUNDING INFORMATION

No funding was received.

## ETHICS STATEMENT

All study participants provided signed informed consent in the local language (Kinyarwanda) prior to data collection. We received ethical approval from the Rwanda National Ethics Committee (Kigali, Rwanda, No.: 48/RNEC/2022) as well as from IntraHealth International's Institutional Review Board (Chapel Hill, NC 27517, United States, No.: 21006).

## Data Availability

The datasets for the current study are available from the corresponding author on request.
